# A rare case of adult H3 K27-altered diffuse midline glioma with multifocal cerebrospinal fluid disseminated disease

**DOI:** 10.1016/j.radcr.2025.11.042

**Published:** 2025-12-04

**Authors:** Jackie Chen, Samuel Conyngham, Angela Bayly, Jacqueline McMaster, Jason Trang

**Affiliations:** a Department of Radiology, Westmead Hospital, Sydney, NSW, Australia; b Tissue Pathology and Diagnostic Oncology, Institute of Clinical Pathology and Medical Research, Westmead Hospital, NSW, Australia; c Department of Neurosurgery, Westmead Hospital, Sydney, NSW, Australia

**Keywords:** Diffuse midline glioma, H3 K27-altered, Magnetic resonance imaging, Biopsy, Brainstem, Cerebrospinal fluid dissemination

## Abstract

H3 K27-altered diffuse midline gliomas are a group of highly aggressive glial brain tumors that occur predominantly in children and carry a poor prognosis with limited treatment options. These are most often found in the thalamus, brainstem, or spinal cord. We report a case of a 28-year-old male with 1-month history of headache who was found to have multiple lesions involving the brainstem, cerebellum, pineal gland, and suprasellar region. On magnetic resonance imaging, the lesions demonstrated mild T2/FLAIR hyperintensity, T1 hypo- to isointensity, and rim contrast enhancement with central necrosis. Subtle diffusion restriction was noted across the lesions. Histopathological analysis following stereotactic biopsy confirmed the diagnosis of H3 K27-altered diffuse midline glioma.

## Introduction

H3 K27-altered diffuse midline glioma is a narrowly defined group of high-grade pediatric diffuse gliomas introduced in the WHO classification of CNS tumors in 2016. It primarily occurs in children and is characterized molecularly by K27 alterations in the histone H3 gene, and typically arises in midline locations including the thalamus, brainstem, or spinal cord [1]. Its nomenclature was revised from “H3 K27M-mutant” to “H3 K27-altered” in the 2021 revision of the WHO classification to recognize alternative mechanisms of pathogenesis in these tumors [2].

It is an important focus of research as it accounts for approximately 10-20% of pediatric brain tumors [3] and 10-15% of brain tumor-related deaths in children [4]. Historically, pathological diagnosis has been challenging due to the difficult to access location in the brainstem or thalamus. Recent studies have demonstrated that stereotactic biopsy is safe and diagnostically accurate for brainstem lesions [5,6], allowing for more histopathological analysis of this entity.

H3 K27-altered diffuse midline gliomas are characterized by a substitution of lysine with methionine at position 27 in histone H3 (H3 K27M) or overexpression of the EZHIP gene [7]. It is classified into four subgroups by the WHO classification, however clinical and molecular heterogeneity suggests that there are additional molecular alterations that may drive tumor phenotype and predict tumor response to treatment [7].

Despite advances in genetic understanding, survival remains poor with Ostrem et al. reporting 1-year survival of 55.9% and median survival of 13.4 months from diagnosis [8]. Currently, radiation therapy remains the only treatment modality that has demonstrated a modest survival benefit [9]. Chemotherapy has historically shown limited efficacy, and ongoing clinical trials are investigating novel chemotherapeutic and immunotherapeutic approaches including vaccination [9].

We present a case of H3 K27-altered diffuse midline glioma with unusual multifocal dissemination. The atypical distribution created significant diagnostic uncertainty on imaging and clinical evaluation, with definitive diagnosis achieved only through stereotactic biopsy.

## Case report

A 28-year-old male presented to his general practitioner with a 1-month history of frequent intermittent headaches lasting 2 to 3 hours. The headaches were most severe in the morning and were associated with blurred vision, dizziness, and intermittent right sided paresthesia to the extremities. He had no significant past medical history. An outpatient magnetic resonance imaging (MRI) scan demonstrated multiple midbrain lesions, and he was urgently referred to the Emergency Department for further imaging and Neurosurgical consultation.

On examination he had a right temporal hemianopia, as well as Parinaud’s syndrome with convergence-retraction nystagmus, upwards gaze palsy, and diminished pupillary light reactivity. There were no other neurological deficits on examination of his cranial nerves or extremities. Further workup included MRI of the brain and whole spine with gadolinium contrast to better characterize the lesions, and a CT of his chest, abdomen, and pelvis to assess for any evidence of a primary malignancy.

## Imaging findings

### MRI findings

MRI brain demonstrated multiple lesions located within the midbrain, suprasellar region, cerebellum, and pineal gland.

**Fig. 1:**
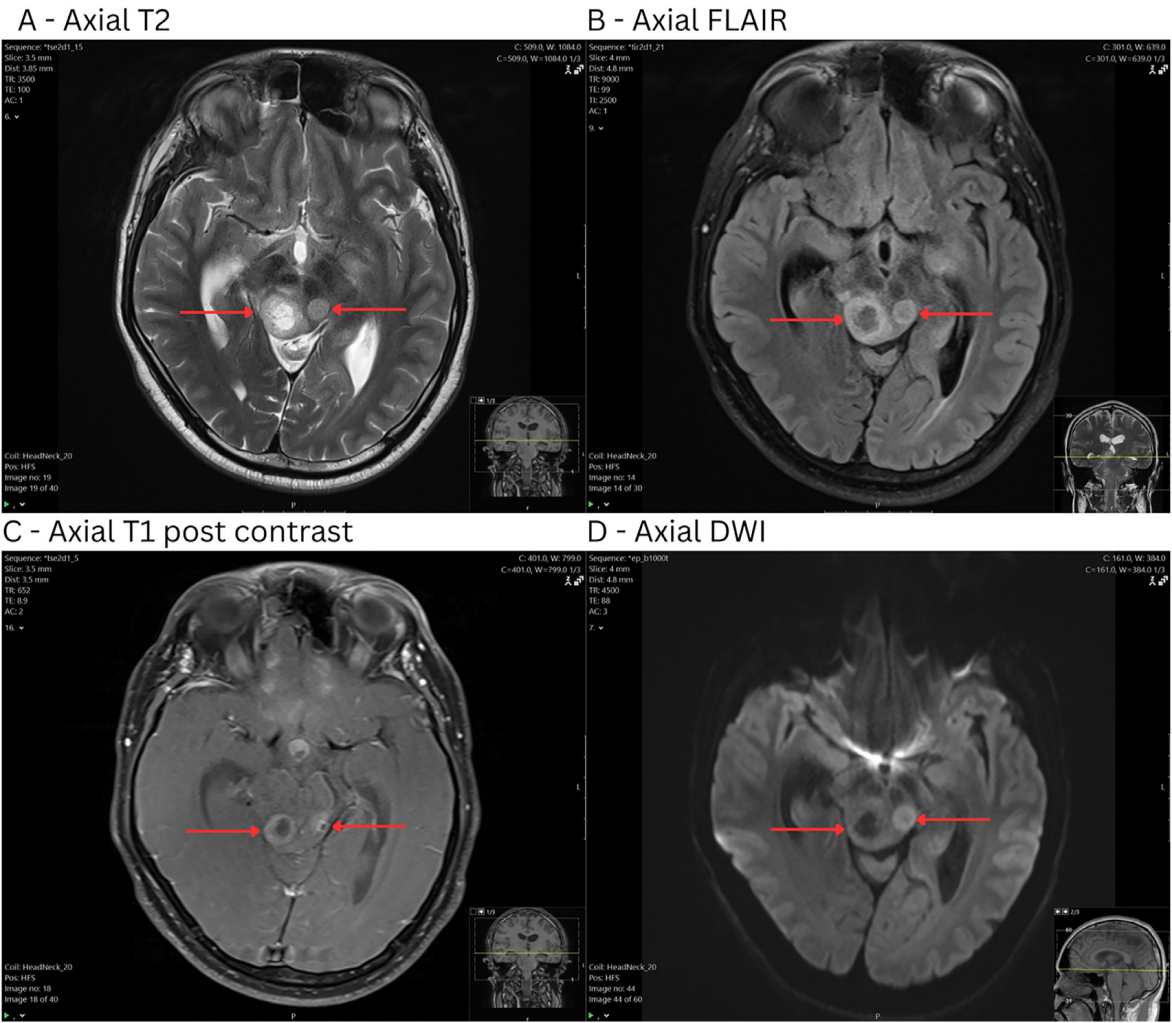
MRI images of midbrain lesions including: (A) Axial T2, (B) Axial FLAIR, (C) Axial T1 post contrast, (D) Axial diffusion-weighted images. 17 × 16 × 16 mm ovoid expansile mass in the right mid brain tectal plate, with a thick continuous peripheral rind of mildly T2 hyperintense, mildly T1 hypointense, avidly enhancing tissue with central nonenhancing, T2 hyperintense, FLAIR hypointense material. Small volume internal low SWI signal consistent with blood products based on the phase imaging. Immediately anteriorly adjacent similar but slightly less avidly enhancing 11 × 10 × 8 mm T2 hyperintense right posterocentral midbrain lesion with a probable small focus of central nonenhancement. Probable subtle diffusion restriction along the medial margin. Small volume internal low SWI signal consistent with blood products.

Focused within the midbrain (Fig. 1) were multiple lesions with mild T2/FLAIR hyperintensity, and T1 hypointensity. The lesions had avid rim enhancement on post contrast sequences with nonenhancing T2 hyperintense, T1 and FLAIR hypointense central components indicating central necrosis. On diffusion-weighted imaging, there were some possible regions of diffusion restriction around the margins of the lesions and facilitated diffusion in the central areas of necrosis. Susceptibility artifact within the lesions was consistent with small amounts of blood products.

**Fig. 2:**
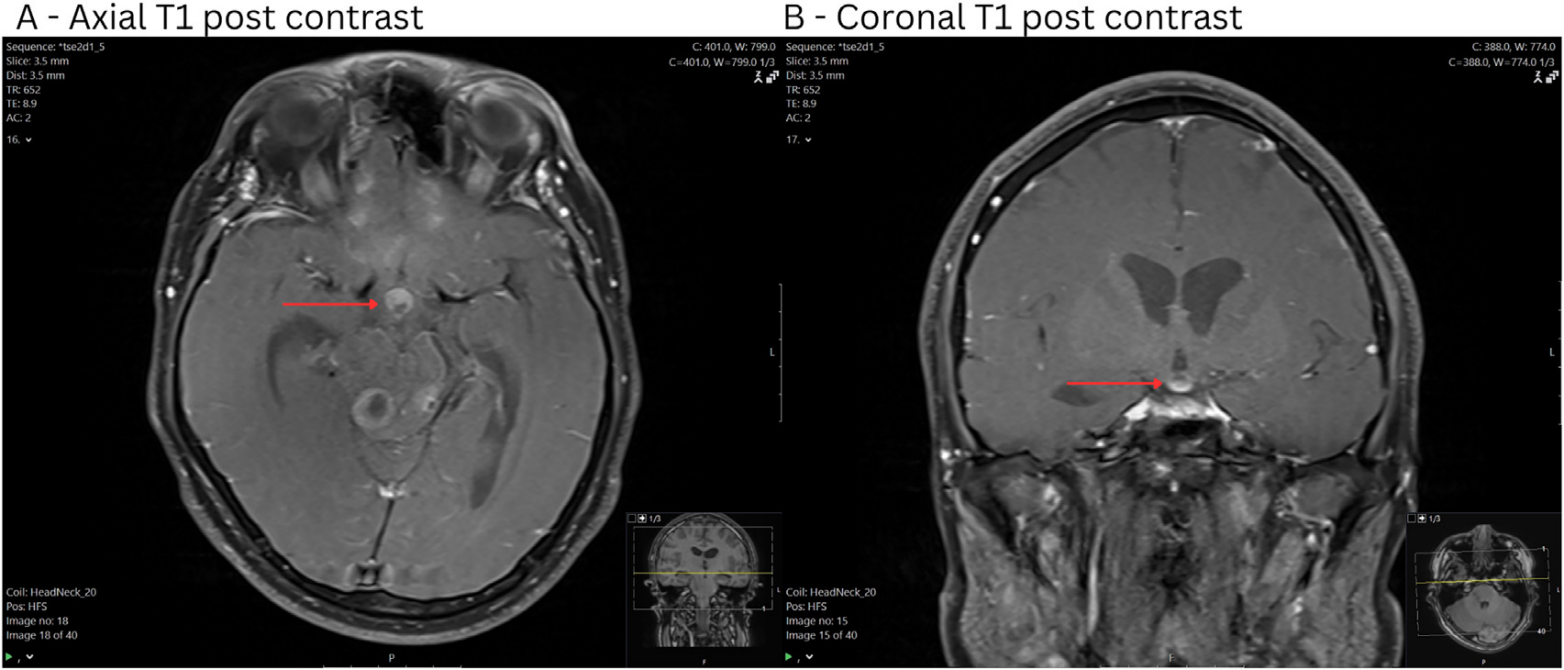
MRI images of suprasellar lesion including: (A) Axial T1 post contrast, (B) Coronal T1 post contrast. Total 10 × 10 × 7 mm heterogeneously enhancing mildly T2 and FLAIR hyperintense, T1 iso- to hypointense suprasellar mass intimately associated with the optic chiasm, appearing separate from the pituitary gland.

Within the suprasellar region (Fig. 2), there was a single lesion demonstrating a similar pattern of T2/FLAIR hyperintensity and T1 hypointensity. It also demonstrated avid rim enhancement with a small central area of necrosis. This lesion was closely associated with the optic chiasm and appeared separate to the pituitary gland.

**Fig. 3:**
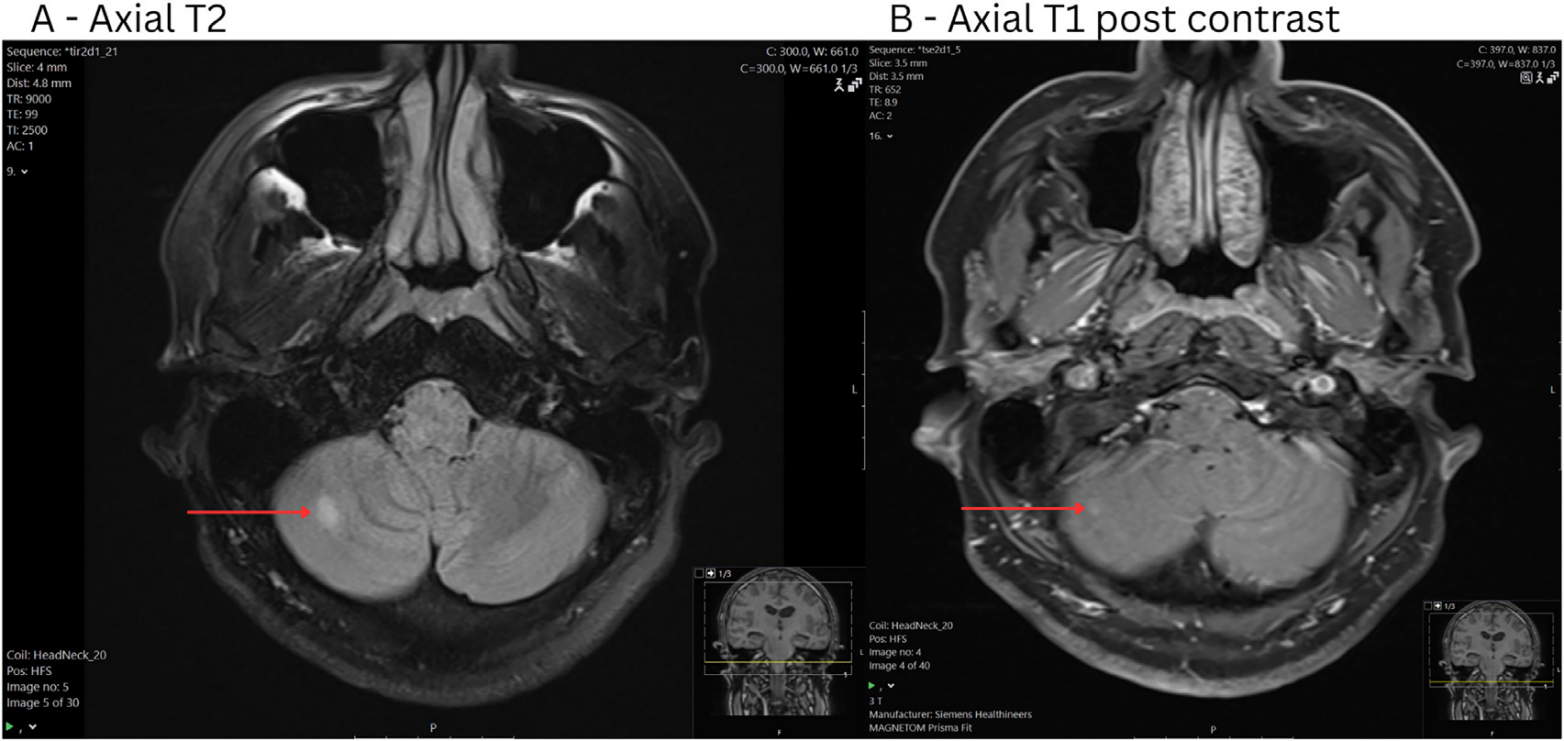
MRI images of a right cerebellar lesion including: (A) Axial T2, (B) Axial T1 post contrast. Total 7 mm subtle enhancing T2/FLAIR hyperintense mass right inferolateral cerebellum. DWI sequence demonstrated diffusion restriction.

Two smaller lesions were seen in the cerebellum (Fig. 3), which demonstrated subtle hyperintensity on T2 and FLAIR sequences. There was no rim enhancement or central necrosis. Diffusion restriction was present.

**Fig. 4:**
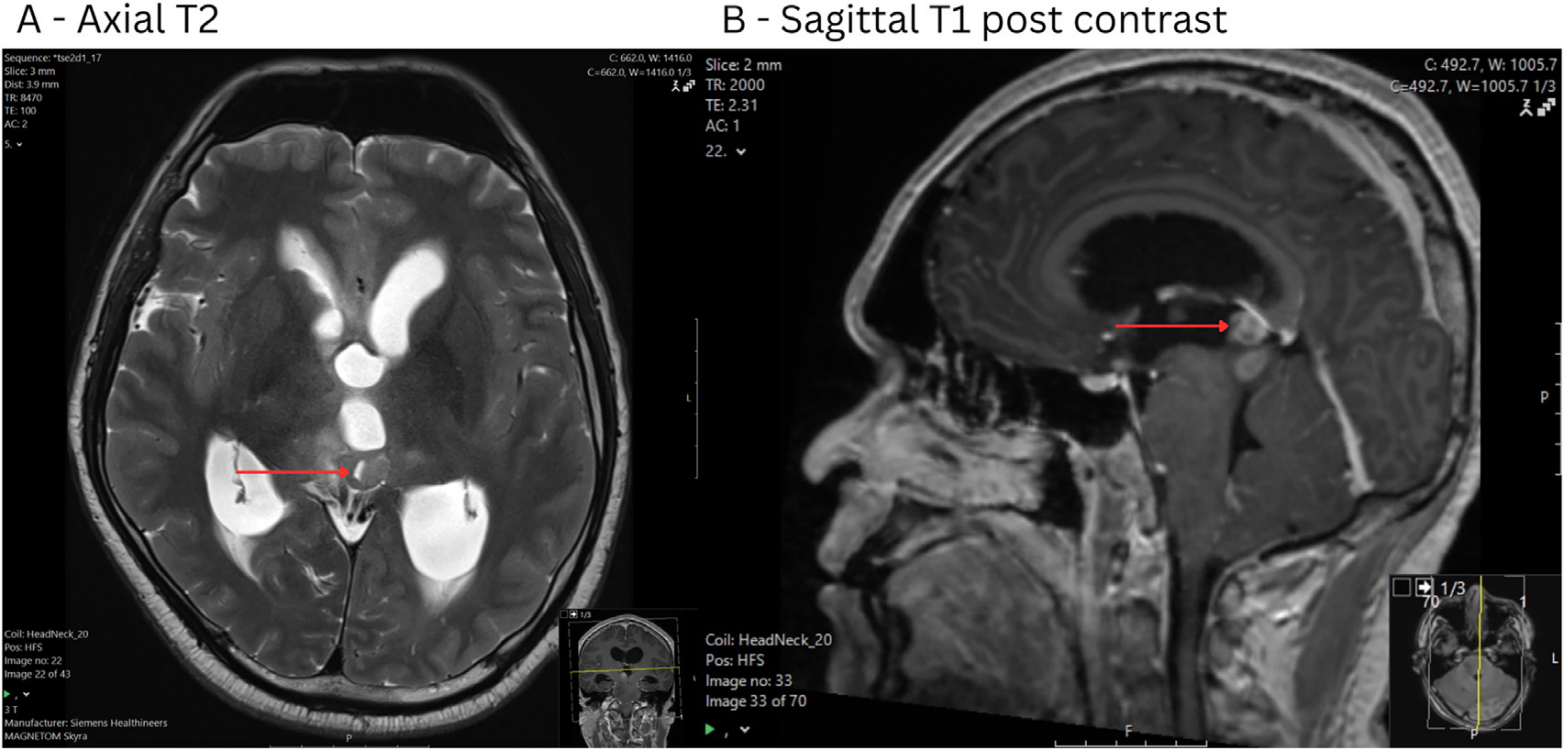
MRI images of a pineal gland lesion including: (A) Axial T2, (B) Sagittal T1 post contrast. This was taken at a later date and the patient developed hydrocephalus. 16 × 11 × 10 mm heterogeneously enhancing mass in the region of the pineal gland, of peripheral mild T2/FLAIR hyperintensity, T1 isointensity and central T2 hyperintensity/nonenhancement. Subtle associated diffusion restriction.

Within or adjacent to the pineal gland (Fig. 4) was a T2/FLAIR hyperintense, T1 isointense lesion which demonstrated rim enhancement and central necrosis. Subtle diffusion restriction was present.

MRI of his whole spine with gadolinium contrast did not demonstrate any abnormal lesions.

### Differential diagnoses

Based on the clinical history and MRI findings, there were a broad range of differentials. This included glial cell neoplasms of the tectal plate and optic chiasm (raising the possibility of neurofibromatosis type 1), a pineal neoplasm with metastases, metastatic disease from a distant source, lymphoma, neurosarcoid, or infection (such as tuberculosis) although less likely. The concomitant pineal gland and suprasellar masses raised the possibility of a malignant germ cell neoplasm but was considered less likely given the patient’s age.

### Further investigation

CT of the chest, abdomen, and pelvis, and whole-body FDG-PET scan demonstrated no evidence of a primary site of malignancy outside of the brain. Bronchoscopy was performed for a region of right upper lung consolidation which demonstrated no evidence of tuberculosis. Endocrinological work up of the pituitary lesion was completed which was unremarkable. He was also investigated for possible adrenal insufficiency which was also unremarkable.

The patient’s condition worsened during admission, with worsening hydrocephalus causing a decrease in his level of consciousness and declining Glasgow Coma Scale (GCS) score. An external ventricular drain (EVD) was emergently inserted, and a sample of cerebrospinal fluid was taken for analysis. There was no infection, and cytological analysis was unremarkable. Testing of serum and CSF germ cell markers including alpha-fetoprotein (AFP) and beta-human chorionic gonadotropin (beta-hCG) were both negative.

Following EVD placement, his GCS score and level of consciousness improved.

### Pathological diagnosis

**Fig. 5:**
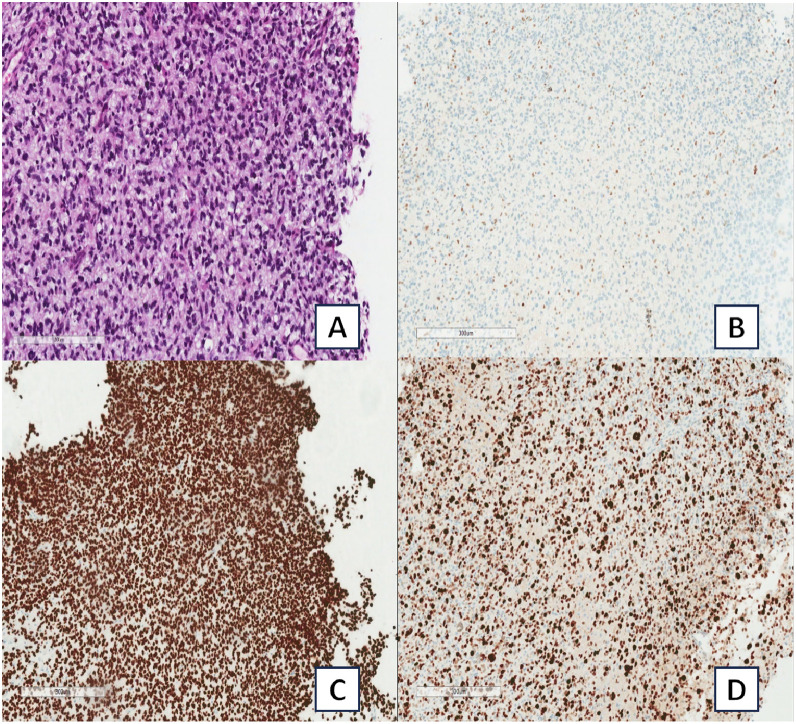
Right cerebellar biopsy. (A) High-grade infiltrating glial cells with scattered mitotic figures. (B) H3K27ME3 demonstrating loss of staining within neoplastic cells. (C) H3K27M showing diffuse nuclear positivity. (D) Ki-67 proliferation index demonstrating high proliferation rate.

Stereotactic biopsy of the right cerebellar lesion was performed to establish a pathological diagnosis. Histopathological and immunohistochemical analyses (Fig. 5) demonstrated a high-grade glioma with frequent mitoses and diffuse H3 K27 positivity with H3K27M E3 loss and overexpression of p53. Additional molecular analysis also detected variants in H3-3A K28M (K27M) and TP53. These molecular findings in conjunction with the midline location were consistent with H3 K27-altered diffuse midline glioma. Given the appearance and distribution of the lesions, the tumor was thought to represent multifocal disease with the likely mechanism of spread via cerebrospinal fluid dissemination.

Consultation with the Radiation Oncology team was made, with a plan to commence radiotherapy and to involve Medical Oncology for consideration of chemotherapy.

### Further progress

**Fig. 6:**
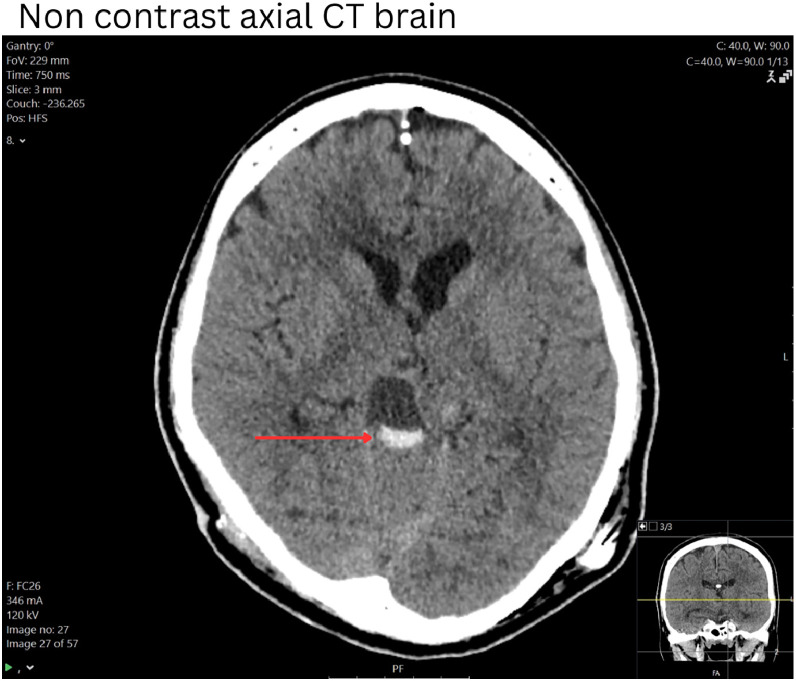
Non contrast axial CT image of the brain demonstrating hemorrhage into the midbrain tectal plate lesions following insertion of a ventriculoperitoneal shunt (not shown). The ventricles are decompressed.

A ventriculoperitoneal shunt was placed for management of ongoing hydrocephalus and to facilitate potential outpatient therapy. Unfortunately, during his admission, his level of consciousness deteriorated from a GCS of 15 to 8 or below. CT of the brain (Fig. 6) demonstrated new hemorrhage into the tectal plate lesion. After a period of close observation without clinical improvement, the decision was made to transition to comfort-focused care given his poor prognosis. The patient passed away several days later.

## Discussion

Diffuse midline gliomas are a high-grade, highly infiltrative tumor that typically originate in the pons, thalamus, or spine. Over 80% of these harbor alterations in the histone H3 gene H3F3A, or less commonly in the related gene HIST1H3B [1]. Despite advances in understanding the molecular pathology of these tumors, the median survival remains low at 13.4 months from diagnosis [8].

Imaging is a critical component in the diagnosis of diffuse midline gliomas and guides further investigation and treatment. A retrospective study by Wang et al. of 120 diffuse midline gliomas, with 61 being H3 K27-altered, found that the most common locations were the brainstem (13.3%), spinal cord (20%), and thalamus (10.8%) [10]. Other locations included the cerebellum (1.7%), corpus callosum (2.5%), lateral ventricle (0.8%), frontal lobe (0.8%) and temporal lobe (0.8%) [10]. In this cohort, there were no lesions in the suprasellar region or the pineal gland. One case report has described synchronous lesions in the pineal gland and suprasellar region [11], and another reported a lesion in the hypothalamus [12].

Classically, diffuse midline glioma has been described as an infiltrative expansile masses with T1 hypointensity, T2 hyperintensity, and minimal contrast enhancement on MRI [13]. Research into the imaging characteristics has demonstrated considerable variability in imaging characteristics which can make radiological diagnosis challenging. Diffuse midline gliomas demonstrate heterogeneous T2/FLAIR signal [14], variable contrast enhancement, and variable central necrosis. A retrospective review of 24 cases of pediatric H3 K27-altered diffuse midline glioma by Aboian et al. reported contrast enhancement in 50% of thalamic lesions and 67% of pontine lesions [15]. Cystic components or necrosis were reported in 63% of patients, with 50% of thalamic lesions demonstrating necrosis [15].

These tumors often exhibit mild diffusion restriction due to increased cellularity similar to glioblastomas [16]. Compared with midline glioblastomas, diffuse midline gliomas exhibit less oedema [16]. There is growing interest in artificial intelligence-based multiparametric radiomic models to predict the presence of the H3 K27M mutation in diffuse midline gliomas based on MRI imaging [17].

This case demonstrated an unusual distribution of lesions with a broad range of differential diagnoses. The lesions involved the midbrain, cerebellum, pineal gland, and suprasellar region. On MRI they were mildly T2/FLAIR hyperintense, and T1 hypo- to isointense. There was variable contrast enhancement, and variable presence of central necrosis. The lesions demonstrated subtle diffusion restriction.

Differentials for this included: primary CNS neoplasm, metastases, lymphoma, neurosarcoid, and infection. A malignant germ cell neoplasm was considered unlikely and ruled out based on negative AFP and beta-HCG testing and the patient’s age. Given the diagnostic uncertainty, treatment options could not be explored until pathological confirmation was obtained.

The consensus was that this likely represented a multifocal [18] H3 K27-altered diffuse midline glioma with multifocal cerebrospinal fluid disseminated disease. Only one other case report in the literature has described synchronous lesions in the pineal gland and suprasellar region [11]. This highlights the importance of considering diffuse midline glioma in the differential diagnosis of young adult patients with multiple synchronous brain lesions in anatomically distinct regions.

## Limitations

This case report has several limitations. There was a broad set of differentials proposed for the cause of these lesions, which owes to the variable clinical and radiological presentation of diffuse midline gliomas. The patient unfortunately passed away in hospital prior to any oncological treatment and could not be followed up as a result.

## Conclusion

We present a case of H3 K27-altered diffuse midline glioma in a 28-year-old male with unusual multifocal disease from likely cerebrospinal fluid dissemination to the cerebellum, pineal gland, and suprasellar regions. This case highlights the need to consider diffuse midline glioma as a differential in a young adult patient with multiple supratentorial and infratentorial lesions. Despite advances in molecular characterization, the prognosis remains poor, emphasizing the need for continued research into novel therapeutic approaches.

## Patient consent

Written informed consent was obtained from the patient’s next of kin for publication. This case report was approved by the Western Sydney Local Health Disctrict Research Ethics Committee for submission and publication.
